# Neurosurgical Treatment and Outcome of Pediatric Skull Base Lesions: A Case Series and Review of the Literature

**DOI:** 10.3390/children10020216

**Published:** 2023-01-26

**Authors:** Ladina Greuter, Tim Hallenberger, Raphael Guzman, Jehuda Soleman

**Affiliations:** 1 Department of Neurosurgery, University Hospital of Basel, 4053 Basel, Switzerland; 2 Department of Pediatric Neurosurgery, University Children’s Hospital Basel, 4056 Basel, Switzerland; 3 Faculty of Medicine, University of Basel, 4056 Basel, Switzerland

**Keywords:** skull base lesions, craniopharyngioma, pediatric neurosurgery, skull base tumor, neuro-oncology, rare disease

## Abstract

Introduction: Pediatric skull base lesions occur rarely and are of various etiologies. Traditionally, open craniotomy has been the treatment of choice; however, nowadays, endoscopic approaches are increasingly applied. In this retrospective case series, we describe our experience in treating pediatric skull base lesions and provide a systematic overview of the literature on the treatment and outcome of pediatric skull base lesions. Methods: We conducted a retrospective data collection of all pediatric patients (<18 years) treated for a skull base lesion at the Division of Pediatric Neurosurgery, University Children’s Hospital Basel, Switzerland, between 2015 and 2021. Descriptive statistics and a systematic review of the available literature were additionally conducted. Results: We included 17 patients with a mean age of 8.92 (±5.76) years and nine males (52.9%). The most common entity was sellar pathologies (n = 8 47.1%), with craniopharyngioma being the most common pathology (n = 4, 23.5%). Endoscopic approaches, either endonasal transsphenoidal or transventricular, were used in nine (52.9%) cases. Six patients (35.3%) suffered from transient postoperative complications, while in none of the patients these were permanent. Of the nine (52.9%) patients with preoperative deficits, two (11.8%) showed complete recovery and one (5.9%) partial recovery after surgery. After screening 363 articles, we included 16 studies with a total of 807 patients for the systematic review. The most common pathology reported in the literature confirmed our finding of craniopharyngioma (n = 142, 18.0%). The mean PFS amongst all the studies included was 37.73 (95% CI [36.2, 39.2]) months, and the overall weighted complication rate was 40% (95% CI [0.28 to 0.53] with a permanent complication rate of 15% (95% CI [0.08 to 0.27]. Only one study reported an overall survival of their cohort of 68% at five years. Conclusion: This study highlights the rarity and heterogeneity of skull base lesions in the pediatric population. While these pathologies are often benign, achieving GTR is challenging due to the deep localization of the lesions and eloquent adjacent structures, leading to high complication rates. Therefore, skull base lesions in children require an experienced multidisciplinary team to provide optimal care.

## 1. Introduction

Pediatric skull base lesions are rarely encountered and can be of various etiologies [1,2]. The most common pediatric skull base lesions described are craniopharyngiomas, benign but locally aggressive tumors often causing vision and endocrine deficits [3,4]. Their clinical presentation varies, and the treatment of these often-complex lesions can pose several challenges. Traditionally, open craniotomy has been the treatment of choice; however, with the recent advantages of endoscopic surgery, new approaches have become available. Endonasal transsphenoidal endoscopic approaches have been successfully applied with the advantage of being minimally invasive; however, they come with limitations, especially due to small nares, narrow inter-carotid distance, and development of pneumatization of the air sinuses in the pediatric population [3,4,5,6]. In cases of cystic craniopharyngioma causing mass effect by protruding into the third ventricle, there is the possibility of a neuroendoscopic transventricular approach with cyst fenestration and subsequent proton therapy [7]. Due to the complex anatomy and multitude of treatment options, a multidisciplinary approach involving pediatric and skull base neurosurgeons, together with craniofacial or otorhinolaryngology (ENT) surgeons, as well as pediatric oncologists and radio-oncologists, is required. Other skull base lesions commonly present in adults, such as skull base meningiomas (fronto-basal, middle fossa, cerebellopontine angle), and vestibular schwannomas are rare in children. Therefore, the literature on pediatric patients is sparse [8]. If they occur, they can be associated with syndromes such as neurofibromatosis type 2 (NF-2) or a history of previous radiation, which should be ruled out if the suspicion is raised [9]. On the contrary, bony lesions/neoplasia of the skull base, such as fibrous dysplasia in young children, can threaten patients’ eyesight and lead to facial deformities, while the growth and thickening of the bone usually come to a standstill after puberty [10]. Since overall skull base lesions remain rare in childhood, the prevalence, treatment, and outcome are mainly described as case reports or small case series.

In this retrospective case series, we describe our experience treating pediatric skull base lesions with either open or endoscopic approaches or a combination thereof. In addition, we provide an overview of the literature on the treatment and outcome of pediatric skull base lesions.

## 2. Materials and Methods

This retrospective case series includes pediatric patients (<18 years) undergoing either microsurgical and/or endoscopic surgery for a skull base lesion. Skull base lesions were defined as purely solid or solid-cystic lesions arising either from the bony skull base, dura, or the pituitary region/Rathke’s cleft. We did not include any arachnoid cysts as they are not solid lesions and mostly require other treatment strategies. The included patients were treated at the Department of Pediatric Neurosurgery, University Children’s Hospital Basel, Switzerland, between 1 January 2015 and 31 December 2021. All surgeries were carried out or supervised by a fellowship-trained senior pediatric neurosurgeon (R.G. and/or J.S.). The study was approved by the local ethics committee (EKNZ 2022-00092), and due to the study’s retrospective nature, patient consent was waived. All data were extracted retrospectively from the secure electronic patients’ database and surgical logbooks. Baseline characteristics such as age, gender, anatomic location of pathology, symptoms, neurologic deficits at admission, and surgical approach were collected.

Outcome measurements were the extent of resection (divided into gross total resection (GTR), subtotal resection (STR), and biopsy (BX)), histopathological diagnosis including WHO grading, length of stay (LOS) in days, if patients went to rehabilitation, clinical outcome (complete recovery, significant improvement, mild improvement, unchanged, mild worsening, significant worsening) at last follow-up, transient (≤90 days) and permanent (>90 days) postoperative morbidity, recurrence or progression of the disease, time to the disease (progression-free survival (PFS)), and surgical treatment due to complications or due to progressive/recurrent disease. WHO grading was based on the current classification at the time of diagnosis. Molecular pathology was not consecutively performed. Good clinical outcome was defined and grouped together as mild/significant improvement or complete recovery. GTR was defined as a complete resection without any residual tumor detected on postoperative imaging, while STR was defined if any size of residual tumor was detected on postoperative imaging. Further, we assessed if patients received either chemo- or radiotherapy after their surgical intervention.

Before treatment, every case was discussed by a multidisciplinary team (MDT) within the scope of our institutional pediatric neuro-tumor-board, including pediatric neurooncologists, pediatric radio-oncologists, pediatric neuroradiologists, pediatric neurologists, and pediatric neurosurgeons. Depending on the specific pathology, skull base surgeons (from the adult neurosurgical department), pediatric endocrinologists, and/or an adult ENT surgeon joined the MDT discussion. All patients received preoperative magnetic resonance imaging (MRI), and if an endonasal endoscopic approach was considered or in case of a bony process, patients received a pre-operative computed tomography (CT) scan as well, to better assess the bony structure and pneumatization of the sinuses. Endonasal endoscopic approaches were usually carried out with colleagues from the adult skull base neurosurgical team and/or an adult ENT surgeon. After surgery, all patients received a postoperative MRI scan within 48 h to assess the extent of resection. All patients with a sellar/suprasellar lesion underwent preoperative endocrine work-up either as outpatients or upon admission. Perioperative cortisol replacement with hydrocortisone was installed, and cortisol levels and urinary output were measured postoperatively. Patients were routinely seen in the pediatric neurosurgical outpatient clinic within 2–6 weeks. Mean follow-up after surgery was 19.76 months (95% CI [10.6, 29]), ranging from 1–50.6 months.

### 2.1. Literature Review

We conducted a review with the following search string in PubMed from its inception until the end of January 2022: (‘Skull base’ [Title/Abstract] OR skull base [Title/Abstract]) AND (pediat* [Title/Abstract] OR children [Title/Abstract] OR child* [Title/Abstract]) AND (tumor [Title/Abstract] OR lesion [Title/Abstract] OR pathology [Title/Abstract]). We only included papers that reviewed more than one single pathology in children and adolescents (<18 years), regardless of the surgical approach (either open or endoscopic or both) and written in English. Case reports, papers focusing on craniometrics only, and technical notes were excluded.

### 2.2. Statistical Analysis 

We calculated descriptive statistics presenting a mean with standard deviation (± SD) or 95% confidence interval (CI) for continuous data and median with interquartile range (IQR) for categorical data.

For analysis of the review, mean weighted rates (complication, mortality rates) and 95% confidence interval (CI) were calculated using the dmetar package for R statistical software [11]. All statistical analyses were carried out using the R statistical software (R Foundation for Statistical Computing, Vienna, Austria, version 4.0.3, 2020). A value of *p* < 0.05 was considered significant.

## 3. Results

### 3.1. Baseline Demographics

We included 17 patients with a mean age of 8.92 (±5.76) years and nine males (52.9%). The most common symptom leading to diagnosis was visual deficits (n = 5, 29.4%), of which four patients (23.5%) had a decrease in visual acuity and one (5.9%) suffered from a visual field deficit. These were followed by endocrine deficits in four children (23.5%, Table 1). Three children (17.6%) suffered from preoperative panhypopituitarism, while one (5.9%) presented with a single hormone axis deficit. Three children (17.6%) only presented with headaches and no other deficits, while three patients (17.6%) had a visible swelling. Motor deficits occurred in one patient (5.9%), which was a clival chordoma compressing the spinal cord resulting in a hemiparesis. One patient (5.9%) had no symptoms, and his diagnosis of an anterior skull base echhordosis physalifora was incidentally detected. The most common location of pathology was sellar/suprasellar in eight patients (47.1%), followed by the anterior skull base in four cases (23.5%).

**Figure 1:**
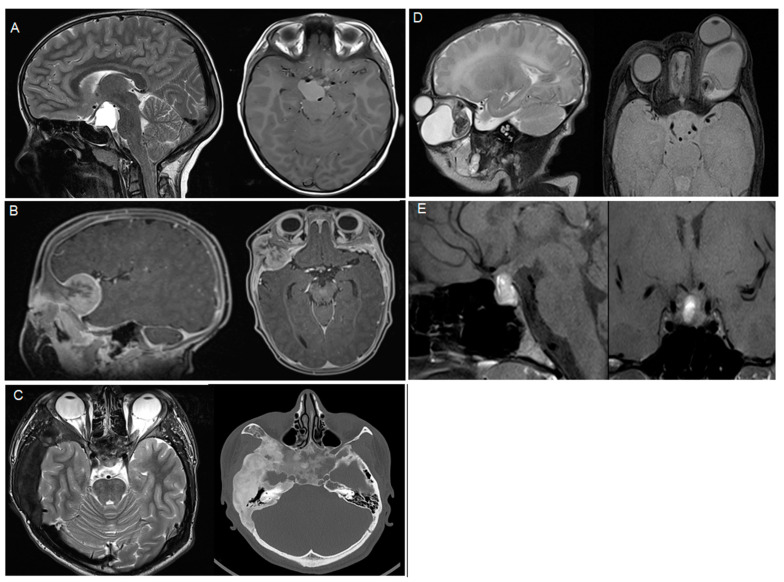
Representative imaging of the included pathologies, (**A**) adamantinomatous craniopharyngioma, (**B**) melanotic neuroectodermal tumor, (**C**) fibrous dysplasia, (**D**) teratoma, (**E**) prolactinoma.

Craniopharyngiomas (all adamantinomatous) were the most common pathology (n = 4, 23.5%), while Rathke’s cysts, osteopetrosis, dermoid cysts, and teratomas were the second most common pathologies (n = 2, 11.8%, each, Table 1). An overview of the pathologies included, with representative imaging, is shown in Table 1 and Figure 1.

### 3.2. Surgical Treatment, Clinical Outcome, and Surgical Morbidity

Just over half of all patients underwent endoscopic surgery (n = 9, 53.0%) of which eight (47.0%) were endonasal procedures and one (5.1%) was a transventricular neuroendoscopic procedure. The neuroendoscopic procedure to fenestrate a cyst was performed in a craniopharyngioma patient. Six patients were treated by pterional approach (35.3%) of which three (17.6%) additionally underwent clinoidectomy to decompress the optic nerves, and two had an extension to an orbito-zygomatic approach (Table 1).

GTR was achieved in half of all patients (n = 10, 58.8%), while in six (35.3%) patients, STR was achieved on postoperative imaging, and one patient with a craniopharyngioma (5.9%) underwent a neuroendoscopic cyst fenestration and biopsy.

The mean length of stay was 8.35 (±3.62) days; one patient (5.9%) was discharged to a rehabilitation facility, while the remaining patients were discharged to their homes. Good clinical outcome was achieved in 13 patients (71.4%), with seven (41.2%) patients making a full recovery. Of the nine (54.1%) patients with preoperative deficits, two (11.8%) showed complete recovery and one (5.9%) partial recovery after surgery. However, in six patients (35.3%), the preoperative symptoms did not recover after surgery three (17.6%) suffered from panhypopituitarism and three (17.6%) suffered from vision impairment. Five patients (29.4%) suffered transient postoperative diabetes insipidus, requiring transient desmopressin therapy. Transient surgical morbidity occurred in six patients (35.3%). The complications were not permanent in any of these patients. Three patients (17.6%) developed an endonasal CSF fistula, of which two (11.8%) required the insertion of a lumbar drain (LD). One patient (5.9%) suffered from a pseudomeningocele after fronto-orbito-zygomatic craniotomy, which was resolved by inserting a ventriculoperitoneal shunt (Table 2). The remaining two complications were a transient left-sided hemi-hyposensibility and a transient sixth nerve palsy after surgery. One patient (5.9%) had a preoperative infected dermoid cyst but no postoperative infectious nor hemorrhagic complications occurred in this cohort. No mortality was observed during follow-up in our cohort.

### 3.3. Adjuvant Therapy

Three patients developed tumor progression (n = 2, 11.8%) or recurrence (n = 1, 5.9%) during follow-up. They all underwent a second tumor surgery. One patient (5.9%) with a melanotic neuroectodermal tumor was postoperatively treated with chemotherapy [12]. Overall mean PFS was 5.96 (95% CI [4.18, 7.74]) months. Three (17.6%) patients were treated with adjuvant radiotherapy, two patients with craniopharyngioma and one with chordoma (Table 2). All patients who received radiotherapy underwent proton beam therapy.

### 3.4. Literature Review

**Figure 2:**
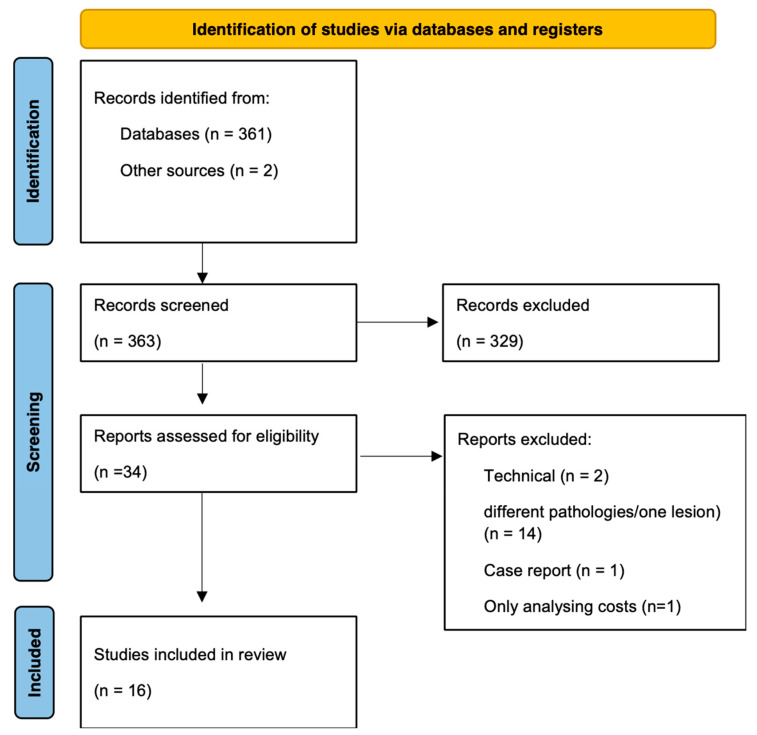
PRISMA flow chart for the review showing the process of the study selection.

We identified 363 reports based on the initial search string. After the initial exclusion of reports based on their titles, 34 abstracts were reviewed, resulting in 16 reports with a total of 790 patients, which were included based on the full-text review (Figure 2). An overview of the included studies is shown in Table 3. Seven studies included solely open approaches or endoscopic approaches, while two studies included open and endoscopic approaches [2,3,4,5,8,13,14,15,16,17,18,19,20,21,22,23]. The mean age of the patients included in the 16 studies was 10.97 years (95% CI [10.8, 11.1]) with 435 (55.1%) male patients. Mean follow-up time amongst all studies was 37.73 (95% CI [36.2, 39.2]) months. The most common pathology described was craniopharyngioma (n = 142, 17.9%, Table 3).

**Figure 3:**
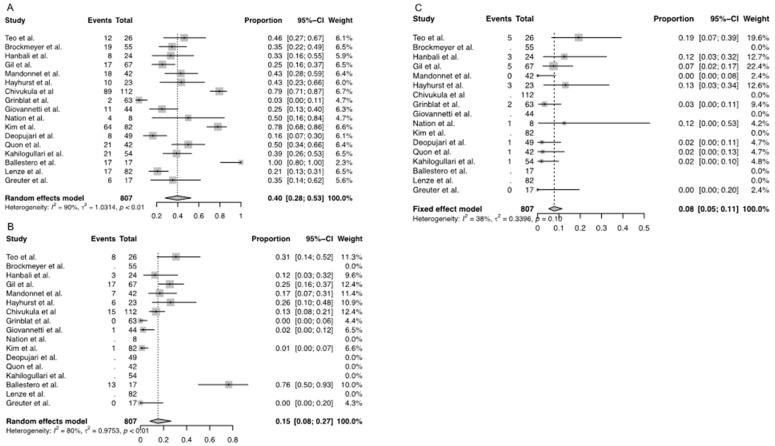
Weighted incidence rate of (**A**) transient complications, (**B**) permanent complications, and (**C**) mortality among all included studies for the review. [2,3,4,5,7,8,13,14,15,16,17,18,19,20,21,22,23].

Overall weighted complication rate was 40% (95% CI [0.28–0.54], Figure 3A), while the overall permanent complication rate was reported by ten studies and was 15% (95% CI [0.08- 0.27], Figure 3B). The overall weighted mortality rate was 8% (95% CI [0.05–0.12], Figure 3C). Mean PFS amongst all the studies included was 36.14 months (95% CI [32.4, 39.9]). 5-year PFS was only reported by two studies, with 70–74% for benign lesions and 43–66% for malignant lesions [13,17]. OS was only reported by one study and was 68% at five years [23].

## 4. Discussion

This study represents one of few case series analyzing pediatric skull base pathologies, their surgical treatment, and outcome. The most frequent symptom leading to diagnosis (n = 5, 29.4%) was vision impairment. The most common location was the sellar/suprasellar region (n = 8, 47.1%), with craniopharyngioma as the most common pathology (n = 4, 23.5%) and 88.2% (n = 15) of benign nature. Endoscopic procedures (including both endonasal endoscopy and neuroendoscopic procedures) were conducted in more than half of all cases (n = 9, 53.0%). Three patients (17.6%) showed progression or recurrence of their disease after treatment. Transient morbidity occurred in six patients (35.5%), while none remained permanent. No mortality occurred in our cohort.

### 4.1. Entity of Skull Base Lesion in Children

Skull base pathologies are a rarity in children, and few case series are available (Table 3). Based on our data and the review conducted, sellar/suprasellar lesions (especially craniopharyngioma) are the most frequent lesions, explaining the high rate of patients presenting with vision impairment or endocrine deficits [3,4,12,16,18,21,24]. A retrospective cohort study by Lenze et al. was the only study to identify non-sellar pathologies within the anterior skull base as the most common skull base lesions in children. However, in their series, only benign tumors were included, and the authors were mainly head and neck surgeons, who usually encounter different pathologies than pediatric neurosurgeons (e.g., mucoceles, fungal sinusitis, and juvenile nasopharyngeal angiofibromas), which probably accounts for the different observation [15].

### 4.2. The Extent of Resection of Various Skull Base Lesions in Children

The traditional approach for craniopharyngioma surgery was to aim for GTR. However, recent literature suggests that especially for craniopharyngioma, the aim of surgery in children is often intended STR instead of GTR to preserve function of the surrounding tissue, mostly hypothalamic function. In case of a progression of the residual tumor, proton beam therapy or radiosurgery was shown to be effective [7,25]. In our series, GTR was achieved in 50% (n = 2) of craniopharyngioma patients, while of the remaining two patients, one received intended STR, and one solely underwent a biopsy. The rate of GTR for craniopharyngioma patients and the whole cohort in our series is lower than seen within the review [2,4,14,16]. However, some of the studies included in the review might have been conducted before this paradigm change and aimed for GTR instead of STR, explaining the higher rates of GTR within these studies [2,19,23].

Surprisingly, no vestibular schwannomas were included in our series, despite vestibular schwannoma being identified as the third most frequent skull base lesion in children according to the review we conducted. In children, most VSs are associated with NF-2, and hearing preservation is the primary goal of surgery. However, a large case series showed that surgery significantly slows down progression [26]. In non-NF-2 associated VSs, the main aim of surgery is GTR while preserving facial nerve function [26,27].

Chordomas arise from the primitive notochord, and in children, one third of them are located at the skull base, mostly at the clivus. They have an infiltrative nature, which makes treatment highly challenging. We report one case of chordoma in our case series, which is comparable to the rate reported by other studies included in our review [2,5,14,28]. The aim of treatment is primary GTR followed by adjuvant radiation therapy [28]. In children, up to 5% of all chordomas can be dedifferentiated, which leads to a high recurrence rate and a low 5-year OS, despite surgery and radiation [28].

Teratomas only account for 0.5% of all pediatric intracranial pathologies but are a relevant differential diagnosis for midline (pineal, sellar region) pathologies. Sellar teratomas occur very rarely, with only few case reports in the literature [29]. Mature teratomas are considered cured after GTR with an excellent long-term outcome survival of 10-year OS of 93% [30,31].

### 4.3. Bony Lesion of the Skull Base in Children

Other common pathologies in ours and other series were bony skull base lesions. We included fibrous dysplasia and osteopetrosis as bony lesions. Craniofacial fibrous dysplasia involves the skull base in 95% and often affects single bone consisting of immature osteoblasts, resulting in the replacement of bony tissue with fibro-osseous connective tissue. Osteopetrosis, on the other hand, is defined by an increase in bone density due to defective osteoclastic resorption [32]. Craniofacial fibrous dysplasia prompts surgery in most patients; however, optic decompression is only recommended in symptomatic patients [10,33]. In this series, the patient was 15 years old and suffered from progressive vision loss, warranting treatment. The timing of surgery in fibrous dysplasia is controversially discussed, and it has been shown that postoperative re-growth can be accelerated in operated children younger than 16 years. After puberty, excessive bone formation usually comes to a halt, and surgical intervention is rarely required in adults [34,35].

### 4.4. Progression/Recurrence of Treated Skull Base Lesions in Children

In our series, three patients (17.6%) presented with a progression/recurrence of the treated lesion. The mean PFS was 5.96 (95% CI [4.18, 7.74]) months, which was shorter than the mean PFS of 36.14 (95% CI [32.4, 39.9]) months reported in the literature [13,16,17,23]. This is most probably due to the multitude of different pathologies included within the different studies as well as different follow-up intervals [13,16,23]. OS is only reported by Hayhurst et al. with an OS of 87% at 5 years [17]. The lack of reported OS could be due to the often benign grading of skull base pathologies, allowing for a long survival after initial diagnosis. More studies with long-term follow-ups would be required to estimate an adequate OS.

### 4.5. Approach to Skull Base Lesions in Children

Endoscopy was the favored surgical approach for pediatric skull base lesions in several case series including ours [15,16,18]. No significant difference regarding complications between open or endoscopic approaches for pediatric skull base approaches has been described in the literature, which is in accordance with our own findings [15]. However, it should be noted that endonasal endoscopy can be challenging, especially in younger children. The main reason is the absent pneumatization of the air sinuses, which can occur as late as the age of 10–12 years [5,36,37]. In case of a poorly pneumatized sphenoid sinus, the surgical landmarks, such as the optico-carotid recess, can be obscured, requiring ample experience in endonasal transsphenoidal endoscopy and technical adjuncts such as image guidance or doppler ultrasound for a successful surgery [38,39]. In our experience, a pediatric neurosurgeon conducts the surgery together with either an experienced skull base neurosurgeon, an ENT surgeon, or both, depending on the underlying pathology and anatomy of the patient. Moreover, it has to be considered that in children under the age of six years, the pyriform aperture distance influencing the transsphenoidal angle is small, making successful endoscopy challenging [16,40]. However, the intercarotid distance in children above nine years and adults is very similar, and once the bony structures are passed, the tumor can usually be removed safely regardless of age [4,18,36]. Furthermore, skull base lesions themselves and surgery thereof before the age of 6–7 can influence nasal and midface growth. There are important preoperative considerations to be discussed with the patient and family, although only a few cases were reported in which patients needed additional corrective measures of the midface [3,41]. To facilitate surgery, smaller adapted endoscopic optics and instruments should be considered in children under the age of nine years [18]. There is no clear age cut-off for transsphenoidal endoscopic surgery in the literature, but due to its challenging nature, it should only be carried out by an experienced team in a multidisciplinary setting [16,18,41].

Even though endonasal transsphenoidal endoscopy is more commonly used, neuroendoscopy via the ventricles is an important approach for skull base lesions extending upwards into the third ventricle, causing hydrocephalus. Especially in pediatric craniopharyngiomas, endoscopic cyst drainage and insertion of a reservoir is a widely accepted treatment strategy [42]. It has the advantage of being minimally invasive with a small risk of blood loss; however, it is often combined with resective surgery and/or radiotherapy at a later stage since resection through the neuroendoscopic approach reaches its limits for large or highly vascularized lesions [43,44].

Open resections are the traditional approach and are still a mainstay of therapy in skull base lesions; however, blood loss especially in small children must be contained, and the re-fixation of the bone flap or skull reconstruction can pose challenges during skull growth later in life. Insufficient bony reconstruction can lead to the formation of pseudomeningoceles or CSF fistulas, while patients receiving a reconstruction with an artificial cranioplasty sometimes require a second surgery later in life to account for their skull growth, and many implants lack osteointegration [12,45]. In general, despite an excellent recovery from surgical complications, preoperative existing deficits, especially vision impairment or panhypopituitarism, marginally improve after surgery, and early diagnosis and treatment are advisable [46,47,48].

### 4.6. Limitations

This retrospective study is subject to all limitations of such a study design. Our mean follow-up time was 16.8 months (95% CI [10.6, 29]), while no patient had a follow-up of five years or more, limiting our ability to provide data on long-term outcomes. The short duration of follow-up is explained by the fact that patients with a craniopharyngioma were mainly followed by endocrinologists or tumors requiring further therapy by the oncologists, and patients with benign lesions and GTR were discharged from our follow-up after 1–5 years, depending on the lesion. In addition, we included a multitude of different pathologies, which have different treatment approaches and prognoses. This heterogeneity of the included patients possibly influenced the different outcome parameters, and other case series which included only a certain group of pathologies are not directly comparable. The number of patients included within this cohort is relatively low, as expected in such rare pathologies in children; therefore, a review was conducted, and a larger number of patients was analyzed. For the review, we only included studies in English and cannot exclude any publication bias of negative unpublished data for this review. Various pathologies were described in the papers included in the review, which could lead to heterogenous results. To provide a broad overview of the literature reflecting the rare and heterogenous nature of pediatric skull base lesions, we included articles in the systematic review that include pathologies which are not observed in our cohort. Hence, only limited recommendations regarding these pathologies are possible. Despite these limitations, this is a well-sized case series of pediatric skull base pathologies and the most up-to-date review regarding this topic.

## 5. Conclusions

This single-center retrospective cohort study highlights the rarity and heterogeneity of skull base lesions in the pediatric population. Craniopharyngioma is the most common skull base lesion in the pediatric population. The review showed an overall PFS of 36.14 months and a mortality rate of 8%.

Although these pathologies are often benign, achieving GTR is challenging due to the localization of the lesions with eloquent adjacent structures or their infiltrative nature, leading to rather low PFS rates and high complication rates. Nowadays, endoscopic surgical approaches are conducted in most cases; however, due to the lack of pneumatization in young children and obscured landmarks, these surgeries can be challenging. Skull base lesions in children require an experienced multidisciplinary team consisting of pediatric neurosurgeons, adult skull base neurosurgeons, ENT surgeons, as well as pediatric neuro- and radiooncologists to achieve optimal care and outcome.

## Figures and Tables

**Table 1:** Demographic Data.

	Overall
n	17
Age (mean (±SD))	8.92 (5.76)
Gender = Male (%)	9 (52.9)
Location (%)	
Anterior Fossa	4 (23.5)
Clivus	1 (5.9)
CCJ/cervical spine	1 (5.9)
Middle Fossa	1 (5.9)
Orbit	2 (11.8)
Sella	8 (47.1)
Pathology (%)	
Craniopharyngioma (WHO I)	4 (23.5)
Rathke’s Cyst	2 (11.8)
Dermoid cyst	2 (11.8)
Osteopetrosis	2 (11.8)
Teratoma (WHO I)	2 (11.8)
Chordoma (WHO I)	1 (5.9)
Echhordosis Physalifora	1 (5.9)
Fibrous Dysplasia	1 (5.9)
Melanotic Neuroectodermal Tumor	1 (5.9)
Prolactinoma	1 (5.9)
Clinical Presentation (%)	
Headaches = yes (%)	3 (17.6)
Vision = yes (%)	5 (29.4)
Motor Deficits = yes (%)	1 (5.9)
Panhypopituitarism = yes (%)	3 (17.6)
Single Hormone deficit = yes (%)	1 (5.9)
Hydrocephalus = yes (%)	0 (0.0)
Surgical Approach (%)	
Transsphenoidal endoscopy	8 (47.1)
Pterional craniotomy ± clinoidectomy	4 (23.5)
Fronto-temporo-orbito-zygomatic	2 (11.8)
Neuroendoscopic cyst fenestration	1 (5.9)
Bifrontal craniotomy	1 (5.9)
Far lateral approach + laminoplasty HWK 2/3	1 (5.9)

Abbreviations: CCJ = cranio-cervical junction.

**Table 2:** Overall surgical outcome.

	Overall
n	17
Extent of resection (%)	
BX	1 (5.9)
GTR	10 (58.8)
STR	6 (35.3%)
Preoperative deficits (%)	6 (35.3%)
Transient DI postop = yes (%)	5 (29.4)
CSF fistula postop = yes (%)	3 (17.6)
Pseudomeningocele = yes (%)	1 (5.9)
Transient Surgical Complications (%)	6 (35.3)
Transient Surgical Complications requiring Surgery (%)	3 (17.6)
Permanent surgical complications (%)	0 (0.0)
Rehabilitation = yes (%)	1 (5.9)
LOS (mean (±SD)), days	8.35 (3.62)
Chemotherapy = yes (%)	1 (5.9)
Radiotherapy = yes (%)	3 (17.6)
Clinical outcome (%)	
Complete recovery	7 (41.2)
Significant improvement	5 (29.4)
Mild improvement	1 (5.9)
Stable	4 (3.5)
Recurrence/Progression = yes (%)	3 (17.6)
Mortality = yes (%)	0 (0.0)

Abbreviations: LOS = length of stay, BX = biopsy, GTR = gross total resection, STR = subtotal resection, DI = diabetes insipidus, CSF = cerebrospinal fluid.

**Table 3:** Results from systematic review of other case series about pediatric skull base lesions.

	Author	Year	N	Surgical Approach	Mean Age/Gender	Three Most Common Pathologies	Extent of Resection & Mean PFS/OS	Overall Complications (n)	Follow-up (Months)	Comments
1	Teo et al. [2]	1999	26	open	10.5 years 18 males	Schwannoma n = 7, 27% Chondrosarcoma/Chordoma n = 3, 11% Esthesioblastoma n = 2, 7% Fibrous dysplasia n = 2, 7% Ependymoma n = 2, 7%	GTR n = 24, 92%	Mortality n = 5 (n = 1 surgical), 19% Complications = 12, 46% CSF leak n = 4, 15% Permanent complications n = 8, 30% (Facial palsy = 4, quadriplegia = 1, panhypopituitarism = 1, dysphagia = 2, blindness =1)	22	
2	Brockmeyer et al. [20]	2003	55	open	9.8 years 30 males	Astrocytoma n = 13, 24% Craniopharyngioma n = 6, 11% Juvenile angiofibroma n = 6, 11% Meningioma n = 3, 5.5% Facial nerve decompression n = 3, 5.5%	NA	Complications = 19, 35% Cranial nerve palsy n = 12, 22% Hemiparesis n = 4, 7% Transient DI n = 3, 5% CSF leak n = 0 Infection n = 0	58	Including astrocytomas and trauma
3	Hanbali et al. [13]	2004	24	open	13.9 years 12 males	juvenile nasopharyngeal angiofibroma n = 4, 17% nerve sheath tumor n = 3, 13% embryonal rhabdomyosarcoma n = 3, 13% desmoid tumor n = 2, 8%	GTR = NA median PFS 84.8 months (95% confi- dence interval, 0–195.8 months)	Mortality n = 3 (non-surgical), 12% Complications = 8, 33% CSF leak n = 1, 4% Transient trigeminal nerve neuralgia n = 1, 4% Transient DI n = 1, 4% Hearing loss n = 1, 4% Neuropathy n = 3, 13% Wound dehiscence n = 1, 4%	35.5 (range 1–102)	Including ENT pathologies
4	Gil et al. [19]	2005	67	open	11 years 39 males	Craniopharyngioma n = 10, 15% Sarcoma n = 9, 13% Chiasmatic glioma n = 8, 12% Juvenile nasopharyngeal angiofibroma n = 8, 12%	GTR = 49, 73% NTR = 6, 9% STR = 12, 18%	Mortality n = 5 (non-surgical), 7% Complications = 17, 25% Panhypopituitarism n = 6, 9% Hemiparesis n = 2, 3% Osteoradio-necrosis of the maxillary complex n = 2, 3% Ascites n = 2, 3% Infection n = 1, 1.5% Visual-field defects n = 1, 1.5% Carotid injury n = 1, 1.5% SIADH n = 1, 1.5% Air embolus n = 1, 1.5% CSF leak n = 0	3 to 60 (average 32.4)	Including gliomas and ENT pathologies
5	Mandonnet et al. [23]	2007	42	open & endoscopically assisted	13.5 years 21 males	Sarcoma n = 7, 17% Rhabdomyosarcoma n = 6, 14% Juvenile nasopharyngeal angiofibroma n = 5, 12%	GTR = 33, 79% STR = 9, 21% Mean PFS 15 months (range 6–144 months)	Mortality n = 0, 0% Complications = 18, 43% Infection n = 6, 14% CSF leak n = 6, 14% Permanent cranial nerve deficits n = 6, 14%	63 (range 0–162)	ENT pathologies
6	Hayhurst et al. [17]	2013	23	open	Median age 7 years 13 males	Rhabdomyosarcoma n = 4, 17% Dermoid n = 3, 13% Neuroblastoma n = 3, 13% Meningioma n = 3, 13% Neuroblastoma n = 2, 9% Schwannoma n = 2, 9% Angiofibroma n = 2, 9%	GTR = 12, 52% Biopsy n = 1, 4% STR n = 7, 30% Debulking n = 3, 13% Progression-free survival was 95% at 1 year Progression-free survival was 68% at 5 years OS 87% at 5 years	Mortality n = 3 (surgical n = 1), 13% Complications = 10, 43% CSF leak n = 2, 9% Meningitis n = 2, 9% Transient 6th nerve palsy n = 1, 4% Facial palsy n = 1, 4% Cerebellar infarction =1, 4% Hydrocephalus, requiring VPS n = 3, 13%	60 (range 6–156)	
7	Chivukula et al. [22]	2013	112	endoscopy	12.7 years (range 2.3–18.0) 85 males	Angiofibroma n = 24, 21% Craniopharyngioma n = 16, 14% Rathke’s cleft cyst = 12, 11%	GTR = 60, 54% NTR = 29, 26%	Complications = 89, 79% CSF leak n = 14, 12.5% Hydrocephalus n = 6, 5% Hematoma n = 3, 3% Permanent DI n = 21, 19% Transient DI n = 20, 18% SIADH n = 3, 3% Panhypopituitarism n = 2, 2% Infection n = 16, 14% Visual deficits permanent n = 1, 1% CN palsy permanent n = 3, 3%	22.7 months	
8	Grinblat et al. [8]	2017	63	open	13.0 (range 1.5–18) years 37 males	cholesteatoma n = 27, 43% Schwannoma n = 14, 22% Granuloma n = 4, 6%	GTR 55, 87% PFS at 3 years 96.9%	Mortality n = 2 (non-surgical), 3% Complications = 2, 3% CSF leak n = 0 abdominal hematoma n = 1, 1.5% fistula n = 1, 1.5%		Only benign, lateral skull base tumors, trauma, hearing related pathologies
9	Giovannetti et al. [18]	2018	44	endoscopy	12.5 years 16 males	Craniopharyngioma n = 12, 27% Pituitary adenoma n = 8, 18% Meningoencephaloceles n = 4, 9% Sphenoidal fibrous dysplasia n = 4, 9%	NA	Complications = 11, 25% CSF leak n = 2, 5% Infection n = 4, 9% DI transient n = 3, 7% Visual deficits transient n = 1. 2% Visual deficits permanent n = 1, 2%	Range 2–36	Including trauma, focused on cephalometrics
10	Nation et al. [5]	2018	8	endoscopy	4.29 years	Chordoma n = 2, 25% Craniopharyngioma n = 1, 12.5% Neuroblastoma n = 1, 12.5% Rathke’s cleft cyst n = 1, 12.5% nasofrontal encephalocele n = 1, 12.5% mesenchymal hamartoma n = 1, 12.5% dermoid cyst n = 1, 12.5%	GTR = 7, 87.5% STR = 1, 12.5%	Mortality n = 1 (non-surgical), 12% Complications = 4, 50% CSF leak n = 0 Endocrine disturbance n = 3, 38% Velopharyngeal insufficiency n = 1, 12.5%	17.4	Only children <6 years
11	Kim et al. [16]	2019	82	endoscopy	11.4 years (range 4–18 years) 36 males	Craniopharyngioma n = 39, 48% Rathke’s cleft cyst n = 15, 18% Pituitary adenoma n = 13, 15%	GTR = 50, 61% STR = 6, 7% Biopsy = 11, 13% Fenestration = 15, 18% median PFS of craniopharyngiomas 19.0 months (range, 9.0–52.0 months)	Complications = 64, 78% Meningitis n = 6, 7% CSF leak n = 2, 2% Hemorrhage n = 1, 1.5% Hypopituitarism = 55. 67%	6.8 (range 1–102)	
12	Deopujari et al. [3]	2019	49	endoscopy	0.5–18 years	Craniopharyngioma n = 22, 45% Pituitary adenoma n = 8, 16% CSF leak repair n = 5, 10% Meningoencephalocele n = 5, 10%	NA	Mortality n = 1 (surgical, ventriculitis), 2% Complications = 8, 16% DI transient n = 4, 8% Steven Johnson syndrome n = 1, 2% CSF leak n = 3, 6%	NA	
13	Quon et al. [4]	2019	42	endoscopy	12.3 years (4–18 years) 28 males	Craniopharyngioma n = 16, 38% pituitary adenoma n = 12, 29% Rathke cleft cyst n = 4, 10% germinoma n = 4, 10%	GTR = 26, 62% STR = 7, 17% Biopsy = 6, 14% Aborted/other = 3, 7%	Mortality n = 1 (coagulopathy, surgical), 2% Complications = 21, 50% CSF leak n = 3, 7% CN deficits n = 3, 7% Endocrine deficits n = 8, 19% Hemorrhage n = 3, 7% Infection n = 2, 5% Graft migration n = 2, 5%	46 months (range 1–120)	
14	Kahilogullari et al. [21]	2020	54	endoscopy	10.4 (range: 1–17) years 33 males	Craniopharyngioma n = 16, 30% Hypophyseal adenoma n = 12, 22% Meningocele n = 5, 9%	NA	Mortality n = 1 (endocrine imbalance), 2% Complications = 21, 39% DI n = 11, 20% CSF leak n = 1, 2% Endocrine problems n = 6, 11% Hemorrhage n = 1, 2% Transient vision loss n = 2, 4%	17 (range: 10–71)	
15	Ballestero et al. [14]	2021	17	open	10.9 years (0.7–17years) 11 males	Schwannoma n = 3, 18% Meningioma n = 1, 6% Chordoma n = 1, 6% Teratoma n = 1, 6% Epidermoid n = 1, 6% Neuroendocrine carcinoma n = 1, 6% Rhabdomyosarcoma n = 1, 6% Juvenile nasopharyngeal angiofibroma n = 1, 6% Hemangiopericytoma n = 1, 6% Myofibroblastic inflammatory tumor n = 1, 6% Fibromyxoid sarcoma n = 1, 6% Crooke’s cell adenoma n = 1, 6% Ossyfing fibroma n = 1, 6% Osteoblastoma n = 1, 6%	GTR = 6, 35% STR = 11, 65%	Complications = 22, 117% CSF leak n = 1, 6% Facial paresis n = 5, 29% Hypacusis n = 5, 29% Oculomotor paresis n = 4, 24% Hydrocephalus n = 3, 18% Visual impairment n = 2, 12% Dysphagia n = 1, 6% Epilepsy n = 1, 6%	72 (range 4.8 to 180)	
16	Lenze et.al [15]	2021	82	open & endoscopy	11.3 (±SD 5.2) years 56 males	Juvenile nasopharyngeal angiofibromas n = 19, 23% Encephalocele n = 15, 18% Fungal sinusitis n = 6, 7%	NA	Complications = 17, 21% CSF leak n = 8, 10% Tracheostomy n = 1, 1% Hemorrhage n = 3, 4% Meningitis n = 5, 6%		Only benign tumors and trauma, ENT pathologies
17	Our series	2022	17	open & endoscopy	8.92 (±5.76) years 9 males	Craniopharyngioma n = 4, 23.5% Osteopetrosis n = 2, 11.8% Dermoid cyst n = 2, 11.8% Teratoma n = 2, 11.8% Rathke Cyst n = 2, 11.8	GTR = 9, 58.8% STR = 6, 35.5% Biopsy =1, 5.9% Overall PFS 5.96 (±3.4) months 2-year PFS 74.1% 2-year OS 100%	Mortality n = 0 Complications n = 6, 35.3% CSF fistula n = 3, 17.6% Pseudomeningocele n = 1, 5.9% Abducens paresis n = 1, 5.9% DI n = 5, 29.4% Hyposensibility n = 1, 5.9% Infection n = 0 Hemorrhage n = 0	16.8 (±16.3)	
	Total (including our data)	1999–2022	807	7 studies open 7 studies endoscopic 3 studies both	10.88 (±2.46) years 435 males	Craniopharyngioma n = 142, 18% Juvenile nasopharyngeal angiofibroma n = 66, 8% Schwannoma/Meningioma n = 33, 4%	GTR = 331, 66% STR = 97, 20% Mean PFS 37.73 (95% CI [36.2, 39.2]) months	Complications n = 344, 42.7% Permanent complications n = 120, 15% Surgical Mortality n = 4, 2% Non-Surgical Mortality n = 18, 7%	36.14 (±21.39)	

Abbreviations: N = number of patients, PFS = Progression free survival, DI = diabetes insipidus, VPS = ventriculoperitoneal shunt, OS = overall survival, SIADH = syndrome of inadequate ADH secretion, AVM = arteriovenous malformation.

## Data Availability

Due to patients’ privacy data is not publicly available.
